# Deaths Attributable to High Body Mass in Brazil

**DOI:** 10.5888/pcd16.190143

**Published:** 2019-10-17

**Authors:** Fabiana M. Rabacow, Catarina M. Azeredo, Leandro F.M. Rezende

**Affiliations:** 1 Universidade Católica Dom Bosco, Desenvolvimento Local, Avenida Tamandaré, 6000, Campo Grande, Mato Grosso do Sul, Brazil; 2 Universidade Anhanguera Uniderp, Campo Grande, Brazil; 3 Curso de Nutrição, Faculdade de Medicina, Universidade Federal de Uberlândia, Minas Gerais, Brazil; 4 Universidade Federal de São Paulo, Escola Paulista de Medicina, Departamento de Medicina Preventiva, São Paulo, Brazil

## Abstract

Our study estimated the proportion of deaths from major noncommunicable diseases (NCDs) that could be prevented in Brazil by reducing population-wide body mass index (BMI) to different counterfactual (optimally theoretical) scenarios. We calculated population-attributable fractions by using BMI data from a representative national survey and relative risks from a published meta-analysis. Reductions in population-wide BMI could prevent 30,715 to 168,431 deaths from NCDs per year in Brazil. Cardiovascular diseases were the most preventable causes of death (5.8%–31.5% deaths prevented). Policies are needed to reduce population-wide BMI in Brazil.

**Summary**

**What is already known about this topic?**

High body mass index (BMI) is among the major modifiable factors to prevent noncommunicable diseases (NCDs).

**What is added by this report?**

We estimated the proportion of deaths from major NCDs that can be prevented in Brazil by reducing population-wide BMI.

**What are the implications for public health practice?**

We found that reductions in population-wide BMI might prevent up to 25.3% of major NCD deaths and 14.9% of all deaths in Brazil. Our findings can help guide public health interventions and policies to prevent NCDs in Brazil.

## Objective

The objective of this study was to estimate preventable deaths from major noncommunicable diseases (NCDs) in Brazil by reducing the population-wide body mass index (BMI) (26.6 kg/m^2^) (1) to the following counterfactual scenarios: 1) theoretical minimum risk exposure level, where adults have a BMI of 22.0 kg/m^2^; 2) reduction in 1.0 kg/m^2^ at population level; and 3) reduction of BMI to levels observed in the Brazilian population in 2002 and 2003 (24.6 kg/m^2^) to show the effect of the continuous increase in BMI over time (2).

## Methods

We obtained BMI data from the National Health Survey, Pesquisa Nacional de Saúde (PNS), conducted in 2013 (2). PNS is the most recent survey using a representative sample of Brazilian adults. Participants were randomly selected in 3 stages: census tracts (primary sample units), households (secondary sample units), and household members aged 18 years or older (tertiary sample units). A total of 62,202 adults were interviewed in the final sample. Both body weight (in kg) and height (in cm) were objectively measured (3). We estimated BMI distribution (mean and standard deviation [SD], prevalence, and 95% confidence intervals [CIs] of overweight [25.0–29.9 kg/m^2^] and obesity [≥30 kg/m^2^]) by sex.

We obtained relative risk (RR) estimates and 95% CIs from the Global BMI Mortality Collaboration meta-analyses (4). RRs were estimated from never-smokers who had no preexisting chronic diseases and excluded the first 5 years of follow-up to reduce confounding and reverse causality (4). We retrieved RR estimates per 5 units of BMI for all-cause, cardiovascular disease, respiratory disease, and cancer mortality.

We retrieved number of deaths from cardiovascular disease (*International Classification of Diseases, Tenth Revision* [ICD-10] codes I00-I99 and R96) (5), respiratory disease (ICD-10 codes J00-J99), and cancer (ICD-10 codes C00-C97 and D00-D48) in Brazil in 2013 by sex and age group from the Brazilian Information System for Mortality (6).

We calculated population attributable fractions (PAF) by sex by using the following equation (7):

PAF = $\frac{\int RR\left(x\right)P\left(x\right)dx-\int RR\left(x\right)P*\left(x\right)dx}{\int RR\left(x\right)P\left(x\right)dx}$

Where *P(x)* is the population distribution of BMI (mean and SD), P*(x) is the counterfactual distribution of BMI, *RR(x)* is the relative risk of NCD associated with BMI (per 1.0 kg/m^2^ increment), and *dx* indicates that the integration occurred with respect to the BMI level. We used a log-logit function to represent each RR value across BMI units (8). We performed data analysis in Stata version 15.0 (StataCorp, LLC).

## Results

Overall mean BMI in Brazil increased from 24.6 kg/m^2^ in 2002 and 2003 to 26.6 kg/m^2^ in 2013. The mean BMI was 27.0 kg/m^2^ (SD, 5.5 kg/m^2^) in women and 26.2 kg/m^2^ (SD, 4.5 kg/m^2^) in men. Approximately 25% of women were obese, and 35% were overweight. The prevalence of obesity in men was 17%, and 40% were overweight.

We estimated that reducing population-wide BMI to a theoretical minimum risk exposure level (22.0 kg/m^2^) could prevent approximately 168,431 deaths per year in Brazil. These deaths represented about 25.3% of major NCD deaths and 14.9% of all deaths that occurred in 2013. Most of these preventable deaths were from cardiovascular disease (106,307), followed by respiratory disease (33,471) and cancer (28,653) (Table).

**Table:** Numbers of Deaths Preventable by Reductions in BMI, by Sex, Counterfactual Scenario, and Causes of Death in Brazil^a^

Outcomes	Total No. of Deaths	Theoretical Minimum Risk Exposure Level^b^		Reduction in 1.0 kg/m^2^ at Population Level^c^		BMI Levels in 2002–2003^d^	
		PAF, %	No. of Preventable Deaths	PAF, %	No. of Preventable Deaths	PAF, %	No. of Preventable Deaths
**Cancer**							
Both	193,936	14.8	28,653	2.5	4,933	5.3	10,366
Men	103,459	13.3	13,764	2.6	2,684	4.7	4,850
Women	90,477	16.5	14,890	2.5	2,249	6.1	5,515
**Cardiovascular disease**							
Both	337,559	31.5	106,307	5.8	19,529	12.4	41,979
Men	176,744	28.9	51,014	5.9	10,438	11.0	19,514
Women	160,815	34.4	55,293	5.7	9,091	14.0	22,465
**Respiratory disease**							
Both	133,124	25.1	33,471	4.7	6,253	10.0	13,376
Men	69,143	22.7	15,690	4.8	3,319	8.8	6,100
Women	63,981	27.8	17,781	4.6	2,934	11.4	7,276
**Major NCD deaths^e^**							
Both	664,619	25.3	168,431	4.6	30,715	9.9	65,721
Men	349,346	23.0	80,468	4.7	16,441	8.7	30,464
Women	315,273	27.9	87,963	4.5	14,274	11.2	35,257
**All-cause mortality**							
Both	1,130,624	14.9	168,431	2.7	30,715	5.8	65,721
Men	635,751	12.7	80,468	2.6	16,441	4.8	30,464
Women	494,873	17.8	87,963	2.9	14,274	7.1	35,257

Abbreviations: BMI, body mass index; NCD, noncommunicable disease; PAF, population attributable fraction; SD, standard deviation.

a Data sources: Brazilian Institute of Geography and Statistics (1), Brazilian Institute of Geography and Statistics (2), Di Angelantonio et al (4), Departamento de Informática do Sistema Único de Saúde (6).

b Theoretical minimum risk exposure level was 22.0 kg/m^2^ (SD, 1.0 kg/m^2^) for both sexes.

c Reduction in 1.0 kg/m^2^ at population level was 25.6 kg/m^2^ (SD, 5.1 kg/m^2^) for both sexes, 25.2 kg/m^2^ (SD, 4.5 kg/m^2^) for men, and 26.0 kg/m^2^ (SD, 5.5 kg/m^2^) for women.

d BMI levels in 2002 and 2003 were 24.6 kg/m^2^ (SD, 4.3 kg/m^2^) for both sexes, 24.5 kg/m^2^ (SD, 3.8 kg/m^2^) for men, and 24.6 kg/m^2^ (SD, 4.8 kg/m^2^) for women.

e Cardiovascular disease, cancer, and respiratory disease mortality.

Reducing population-wide BMI in Brazil to levels observed during 2002 and 2003 (24.6 kg/m^2^) could prevent 65,721 deaths, representing 10.0% of deaths from major NCDs and 5.8% of all deaths. A reduction in 1.0 kg/m^2^ of population-wide BMI could prevent 30,715 deaths, representing 4.6% of deaths from major NCD and 2.7% of all deaths (Table).

## Discussion

Approximately 25.3% of major NCD deaths and 14.9% of all deaths could be prevented each year in Brazil by reducing population-wide BMI. Other scenarios indicated that 4.6% of major NCD deaths could be avoided by reducing 1.0 kg/m^2^ of BMI and 10% of NCD deaths by reducing BMI to levels observed during 2002 and 2003.

The reduction of BMI would have the greatest effect on cardiovascular disease deaths, which account for one-third of all deaths in Brazil (9). The World Health Organization Global Plan for 2025 involves a series of targets to reduce 25% of premature mortality from major NCDs (10), among which is halting the rise in obesity rates. Our study considered more ambitious scenarios of BMI reduction, which can be a challenge. Obesity increase is primarily driven by obesogenic environments. To reverse this trend, some in the scientific community, especially in the fields of nutrition and physical activity, have suggested modifying obesogenic environments through fiscal and regulatory actions (eg, taxation, labeling, marketing of ultraprocessed products) (11).

Our study has limitations. Although BMI is a useful indicator of body fat, it does not differentiate between lean and adipose tissues (12). Furthermore, we used RR estimates from a meta-analysis that included data from 4 continents but not Brazil (4). These RR estimates were not stratified by potential effect modifiers (eg, sex, age). Whether these RR estimates are applicable to Brazilians is unknown. On the other hand, by using RR estimates for never-smokers who had no preexisting chronic diseases and excluding the first 5 years of follow-up, we reduced reverse causation bias and achieved more reliable estimates of deaths attributable to BMI (4).

By reducing population-wide BMI in Brazil, 30,715 to 168,431 deaths per year from NCDs could be prevented. Policies aimed to reduce obesogenic environments are needed to decrease population-wide BMI in Brazil.
